# Enhancing genome investigations in the mosquito *Culex quinquefasciatus *via BAC library construction and characterization

**DOI:** 10.1186/1756-0500-4-358

**Published:** 2011-09-13

**Authors:** Paul V Hickner, Becky deBruyn, Diane D Lovin, Akio Mori, Christopher A Saski, David W Severson

**Affiliations:** 1 Eck Institute for Global Health, Department of Biological Sciences, University of Notre Dame, Notre Dame, IN 46556, USA; 2 Clemson University Genomics Institute, Clemson, SC 29634, USA

**Keywords:** Bacterial Artificial Chromosome, Culicidae, *Culex pipiens/quinquefasciatus*, Lymphatic filariasis, West Nile virus, *Wuchereria bancrofti*

## Abstract

**Background:**

*Culex quinquefasciatus *(Say) is a major species in the *Culex pipiens *complex and an important vector for several human pathogens including West Nile virus and parasitic filarial nematodes causing lymphatic filariasis. It is common throughout tropical and subtropical regions and is among the most geographically widespread mosquito species. Although the complete genome sequence is now available, additional genomic tools are needed to improve the sequence assembly.

**Findings:**

We constructed a bacterial artificial chromosome (BAC) library using the pIndigoBAC536 vector and *Hin*dIII partially digested DNA isolated from *Cx. quinquefasciatus *pupae, Johannesburg strain (NDJ). Insert size was estimated by *Not*I digestion and pulsed-field gel electrophoresis of 82 randomly selected clones. To estimate genome coverage, each 384-well plate was pooled for screening with 29 simple sequence repeat (SSR) and five gene markers. The NDJ library consists of 55,296 clones arrayed in 144 384-well microplates. Fragment insert size ranged from 50 to 190 kb in length (mean = 106 kb). Based on a mean insert size of 106 kb and a genome size of 579 Mbp, the BAC library provides ~10.1-fold coverage of the *Cx. quinquefasciatus *genome. PCR screening of BAC DNA plate pools for SSR loci from the genetic linkage map and for four genes associated with reproductive diapause in *Culex pipiens *resulted in a mean of 9.0 positive plate pools per locus.

**Conclusion:**

The NDJ library represents an excellent resource for genome assembly enhancement and characterization in *Culex pipiens *complex mosquitoes.

## Introduction

*Culex quinquefasciatus *(Say), the southern house mosquito, is a major vector for a number of important human pathogens including West Nile virus and *Wuchereria bancrofti*, the primary global etiologic agent for lymphatic filariasis (LF) [1-3]. It is estimated that more than 1.2 billion people are at risk for infection by parasites causing LF, with 120 million people presently infected [4]. Among these are over 40 million people who suffer from chronic morbidity associated with lymphadema and hydrocele [5]. Despite the availability of effective antihelminthics to treat and prevent infections, the damage to the lymphatic system caused by these parasites is largely irreversible. Although efforts to eradicate LF globally using mass drug administration to human populations in endemic areas were initiated in 2000, the success of these efforts will likely also rely on the implementation of effective mosquito vector control strategies [6]. However, vector control efforts can be hindered by the rapid selection for emergence of insecticide resistance [7]. Consequently, the identification of new targets for insecticides as well as the development of novel vector control strategies is expected to play a large role in the successful control and/or eradication of mosquito-borne diseases [8].

*Cx. quinquefasciatus *and *Cx. pipiens *(L.) are the two most common and geographically widespread species in the *Cx. pipiens *complex, a species complex with nearly worldwide distribution [9]. *Cx. quinquefasciatus *is common in tropical and subtropical regions while *Cx. pipiens*, the northern house mosquito, occupies more temperate regions. Both species are abundant in urban areas where they oviposit in stagnant, and often polluted water. They frequently enter homes and feed on humans during the night, hence the common name of house mosquito. The taxonomic status of this complex has been a subject of debate, and these taxa are sometimes placed within a single species, i.e., *Cx. pipiens quinquefasciatus *or *Cx. pipiens pipiens *[9]. Introgression between these species is common in the United States where hybrids can be found as far south as Louisiana and as far north as Illinois [10-12], yet in South Africa the populations remain largely distinct [13,14]. Females are morphologically indistinguishable, while differences in male genitalia have been used to identify species as well as interspecies hybrids [10-12,15]. Recently, however, PCR assays have been developed to aid in the differentiation of species in this complex [16-19].

Given their medical importance, *Cx. pipiens *complex mosquitoes have garnered considerable attention by the scientific community during the last 100 years [9]. Nevertheless, the current status of contemporary *Cx. pipiens *genetics remains considerably behind that of other important mosquito vectors such as *Anopheles gambiae *and *Aedes aegypti *[20]. The *Cx. quinquefasciatus *(Johannesburg strain) genome sequence was recently determined using the whole genome shotgun (wgs) approach, thus providing a valuable resource for advancing genome studies in this species complex [21]. However, the genome assembly remains highly fragmented and few (~40) of the 3171 supercontigs have been assigned to their respective chromosomes [21].

Bacterial artificial chromosome (BAC) genomic libraries are important resources for the assembly and characterization of complex genomes. They have been utilized for the assembly of numerous genomes including *Drosophila melanogaster *and *An. gambiae *[22,23]. BAC libraries have also been used for the development of genetic markers for non-model organisms [24,25]. Furthermore, BAC clones can be used for positional cloning to help identify and characterize genomic regions of interest [26,27], as well as for construction of BAC-based physical map assemblies [28-31]. These are useful for long-range contiguity and anchoring of wgs draft assemblies as well as targeted re-sequencing for high resolution using BAC pools [32]. The objective of this work was to construct a BAC library with comprehensive coverage of the *Cx. quinquefasciatus *genome, thereby providing a tool to aid in genome assembly, marker development, and gene discovery in *Cx. pipiens *complex mosquitoes.

## Methods

### BAC library construction

High molecular weight DNA was extracted from pupae from the Johannesburg (JHB) strain. This strain was established using individuals from Johannesburg South Africa, and was the strain used in the *Cx. quinquefasciatus *genome project [21]. Pupae were gently homogenized in 1X PBS buffer containing 50 mM EDTA pH 8.0 and 0.1% BME and filtered through one layer of miracloth into 50 mL Falcon tubes. Cells were pelleted by centrifugation in a swinging-bucket rotor (Beckman) at 3,200 rpm for 15 minutes at 4°C. Pellets were washed 2 additional times with PBS and gently resuspended in 1 mL of PBS. The nuclei solution was warmed to 45°C in a waterbath, mixed gently with an equal volume of 1.5% low-melt agarose (Seaplaque) and aliquoted into plug molds (BioRad) using large-bore tips. Protein digestion and plug washing was performed exactly as the methods of Luo and Wing (2003) [33].

*Hin*dIII partial restriction enzyme digestion of DNA, as well as the preparation of high molecular weight DNA fragments was conducted following the procedure of Luo and Wing (2003) [33]. Preparation of the *Hin*dIII cloning-ready single copy pIndigoBAC536 vector from the high copy pCUGIBAC1 plasmid was performed according to Luo *et al*. (2001) [34]. The size selected high molecular fragments were ligated to the vector and transformed into *E. coli *strain DH10B competent cells (Invitrogen, Carlsbad, CA). White recombinant colonies were selected on LB plates containing chloramphenicol, X-Gal and IPTG, and picked robotically using the Genetix Q-bot (Genetix, UK). Recombinant clones were transferred into individual wells of microtiter plates, grown and then stored at -80°C. The BAC library was also gridded onto 10, 11.25 × 22.25 cm filters in high density, double spots (18,432 clones represented per filter) and 4 × 4 patterns.

To estimate the size of the BAC inserts, DNA from 82 randomly selected clones was prepared according to standard alkaline lysis protocol, digested with *Not*I, and separated by pulsed-field gel electrophoresis (PFGE) on a 1% agarose gel under the following conditions: 5-15 sec linear ramp time, 6 V/cm, 14°C in 0.5 × TBE buffer for 15 hours and stained with ethidium bromide. Insert sizes of the clones with endogenous *Not*I sites, evidenced by multiple restriction fragments, were estimated by summing the fragments. Southern blotting was used to confirm that all of the clones were truly *Cx. quinquefasciatus *and are not significantly contaminated by other types of DNA. One gel used for insert size determination was transferred to a positively charged nylon membrane Hybond N^+ ^(GE Healthcare) following the methods of Chomczynski (1992) [35]. BAC vector (pIndigoBAC536) and total *Culex *DNA were used as probes and radiolabeled with the DECAprime™ II kit (Ambion, Inc). The probes were mixed and denatured, and hybridization was carried out overnight at 60°C. The membrane was washed with 1× SSC, 0.1% SDS at 60°C twice for one hour each. The membrane was exposed to a phosphor screen (GE Healthcare) overnight and the image recorded by a Typhoon 9400 imager (GE Healthcare).

### BAC library screening

Screening of the BAC library was generally performed as described by Jiménez *et al*. (2004) [36]. Briefly, we first prepared pools of DNA representing all clones within each of the individual 144 384-well microplates. Plates were initially replicated on LB agar plates containing 12.5 μg/ml chloramphenicol and incubated overnight at 37°C. The plates were then flooded with LB broth containing 12.5 μg/ml chloramphenicol, agitated for 4 h at 37°C and the slurries used to prepare 9.5 ml overnight cultures. These individual plate pool cultures were used for large-scale alkaline lysis DNA extractions [37] and subsequent PCR screening with SSR oligonucleotide primer sets.

A combination of PCR-based plate-pool DNA screening and radiolabeled oligonucleotide probe hybridization was used to screen the NDJ library. Initial PCR-based screening of BAC DNA representing individual plate pools was performed using 29 simple sequence repeats (SSRs) (Table 1) [38-40]. The Primer3 program [41] was used to design primers to amplify regions within a gene on supercontig 3.134 and four genes previously identified as having a role in reproductive diapause in *Cx. pipiens *s.s. (Table 2) [42,43]. PCR reactions were performed in a total volume of 25 μl containing 50 mM KCl, 10 mM Tris (pH 9.0), 0.1% Triton X, 1.5 mM MgCl_2_, 200 μM dNTPs, 5 pmol of each primer (F and R), 25 ng of plate pool DNA and 1 unit of *Taq *polymerase. PCR thermal cycling conditions were 5 min. at 94°C, followed by thirty cycles of 1 min. at 94°C, 1 min. at 60°C, 2 min. at 72°C, and then 10 min. at 72°C for a final extension. The SSR positive plate pools were identified by electrophoresis on 2% agarose gels using ethidium bromide and UV visualization.

**Table 1:** SSR primer sequences.

SSR locus	GenBank accession	SSR Primer Sequence (F/R)	Product size (bp)^a^	# Positive plate pools
C127GAC1	GF102017	GCGTTTGGAGAGTGGAAAAG	307	10
		TGAGTTTTCAGTGCCCTCCT		
C32AC1B	GF110611	AAACGATCGCAATTCGAAAC	242	3
		GTGGCGAACAACATTCACAG		
C32TC1B	GF110612	TCATCGTTCATTCGTTCCAA	179	2
		TGTCATTTTCTGCCTGCATC		
C32TG1	GF102044	CGTGTTTTCCATTGTTGGTG	400	29
		TTGGCTGTGTCAACTGCTTC		
C68ACAT1	GF102032	GGCCTTGCTGAGAAAACTTG	425	1
		CCCAAAATCCAAGCTTCAAA		
C68CA1	GF102033	ATAAAGCGACCAAGGCTCAA	294	7
		GCGAAACCATTCAAAAGCAT		
C68GA1B	GF110613	CACCCCACAGTTAACCCAAC	245	8
		CTCGAGAGATTTGGCCTTTG		
C65AC1	GF102022	GGAGTTGTGCGGTTGAAAGT	305	19
		GCACTGCCTAACGGATCATT		
C65CGC1	GF102023	TCTGGGTACAACCCCGTAAC	221	20
		AGAGAGTGCGCAAAAGCAAT		
C65TG1	GF102021	ACTGCGAAACGCTTACTGCT	302	9
		GTGTGTGGACTGTGGTGGAG		
C474CT1B	GF110614	CCCAAACTTGCCACAAAAGT	290	2
		CTCACTCTCCGTGAACGACA		
C48ATC1	GF102034	CATTTTTCGGGTGGCTTCTA	337	7
		CGAGATCGAAATGATGCTGA		
C48CGA1B	GF110615	GCTTGGGAATCTGAATCTGC	251	4
		ACCTTGCATTCAACGAGCTT		
C48GTT1B	GF110616	GTGGCCACCTGGTTGTAGTT	309	23
		ACCACCGGTAGAACATCTCG		
C175AT1	GF102036	GGACCAAGGGTACGATTTGA	185	14
		CAGACTGGTTAACGGCTTCC		
C175TG1	GF102045	TCAGATCTCCGAGAGGAGGA	295	4
		CTGTCAGGGCCAGATTTCAT		
C134AC1	GF102037	GAAGGTCAGCCACTCAGGC	194	0
		ACAGCTGACTCTCGTCGAC		
C129GT1	GF102038	AAGGTGCAAAACCAAACTGG	377	1
		TGGAGCACAGCCCTACTCTT		
C66CA1	GF102026	CGACTACTGCCCCAATTTGT	213	2
		CACCCTCCCCTACAGACGTA		
C177CA1B	GF110618	AGGGCAATGTTTACGACGAC	293	2
		CTTGCGCCTTAGTCATCCTC		
C177TG1	GF102028	AGCACAAAAAGGCACGATTT	197	6
		TAAACGCAAGTAGGCGGAGT		
C99TGT1B	GF110619	GCAGTGGAGGATTCTGAGGA	358	5
		CAGAACGTTTGGCGAATTTT		
C205CA1	GF102029	CAATGCGCCTTCTGGATTAT	227	3
		CTCGTGATGGCCATTTCTCT		
C205TG1B	GF110620	ATTGCTCAAGTGCTGCCTTT	212	7
		ATGACGACGAAAAACCGAAC		
C139TG1B	GF110621	GGGATCGCTACGTGTTTTGT	265	6
		TCTCGGAATGCCAGTCTTTT		
C446AC2	GF110622	CATACGACGTGGAACAAACG	162	17
		ACGAGGTTGAGGTTGGTGAC		
C446TG1	GF102043	GGAAAGGGGCACTTGTGTAA	397	22
		CGTTTGCTTCTCTTCGAACC		
CxpGT4	AY423738	GTCGTCGCTAACCCTTGTT	146	2
		CGCGATAGTCGGTAATCGT		
CxqTri4	AY958079	CTAGCCCGGTATTTACAAGAAC	121	16
		AACGCCAGTAGTCTCAGCAG		

^a^Predicted amplicon size based on nucleotide sequence in VectorBase [50].

**Table 2:** Primer sequences for genes used in library screening.

Gene	VectorBase gene ID	GenBank accession	SSR Primer Sequence (F/R)	Product size (bp)^a^	# Positive plate pools
CHP*	CPIJ007110	GF110930	CGAGCAGTTCAAACACCAGA	207	12
			GCTTCTTCAGGTTGCTCCAC		
FOXO	CPIJ016794	GF110931	CTGAGCCCAATTCAGTCCAT	187	3
			TCTGCTGTAAAGTCAGCTCGTC		
ILP-1	CPIJ018051	GF110932	AGTCCCTCGGAGGAGTTCAA	163	7
			TCGGCACAGTACTGCTTGAG		
ILP-2	CPIJ018050	GF110933	TCCAGCAGATCTTCGATGC	140	10
			TGTAGATCGGGGAACTCGTC		
ILP-5	CPIJ001698	GF110934	GGTTCCATCACGCAGGAGT	87	23
			GTTGATCCGCTTGTTCGAC		

^a^Predicted amplicon size based on nucleotide sequence in VectorBase [50]. *CHP: Conserved hypothetical protein.

Well position of marker loci within select positive microplates was determined by DNA-DNA hybridization. Four individual clones were identified by probing with P^32^-labeled PCR amplicons (C127GAC1, C65AC1, C99TGT1, and FOXO) and thereafter sized with PFGE. Briefly, microplates representing positive pools were replicated to Colony/Plaque screen hybridization membranes (NEN™, Life Science Products) following Jiménez *et al*. [36]. Hybridizations and radiolabeling of the target clones were conducted following our standard probing procedures [44]. The presence of the marker locus in each of the four clones was confirmed by PCR and UV visualization on 2% agarose gels, as described for plate pool screening.

## Results and Discussion

**Figure 1:**
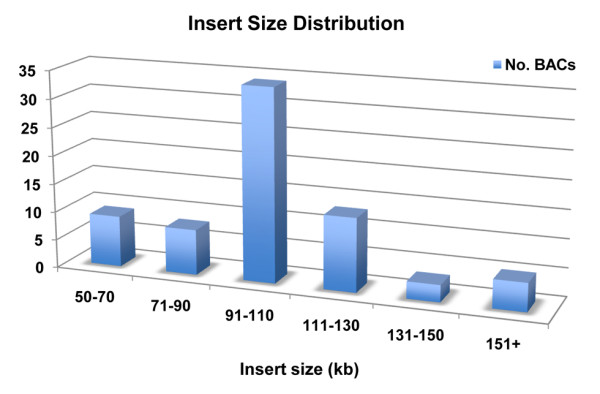
**Insert size distribution of BAC clones in the NDJ library based on pulsed-field gel electrophoresis of 82 randomly selected clones**.

**Figure 2:**
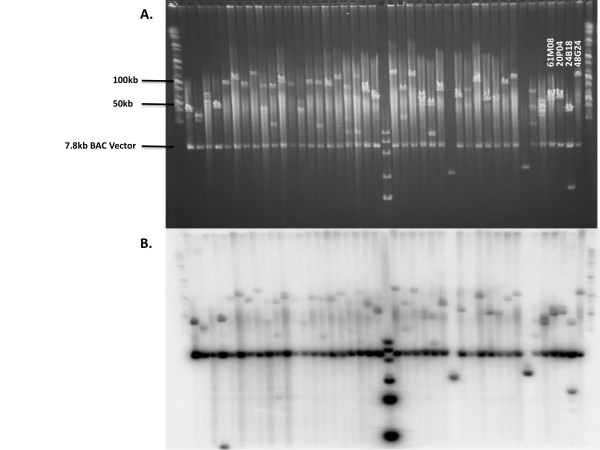
***Not*I digests of *Culex quinquefasciatus *BAC clones**. **A**. Pulsed-field gel electrophoresis of 38 randomly selected clones and 4 clones containing markers used in plate pool screening. **B**. Southern transfer of the BACs from panel A hybridized with a mixture of total *Cx. quinquefasciatus *gDNA and BAC *vector*.

We have constructed a BAC library for *Cx. quinquefasciatus*, an important human disease vector and a major species in the *Cx. pipiens *complex, using high molecular weight DNA extracted from Johannesburg strain pupae and partially digested with *Hin*dIII. The Notre Dame Johannesburg (NDJ) library consists of 144 384-well microplates containing 55,296 clones. *Not*I digestion and pulsed-field gel electrophoresis of 82 randomly selected clones produced fragments ranging from 50 to 190 kb in length (mean = 106 kb) and no empty vectors (Figures 1 and 2). Southern blotting with BAC vector and *Cx. quinquefasciatus *gDNA indicated that the inserts are of *Culex *origin and all BACs appear to be fully digested (Figure 2B). Based on a mean insert size of 106 kb and a genome size of 579 Mbp, the BAC library provides ~10.1-fold coverage of the *Cx. quinquefasciatus *genome.

**Figure 3:**
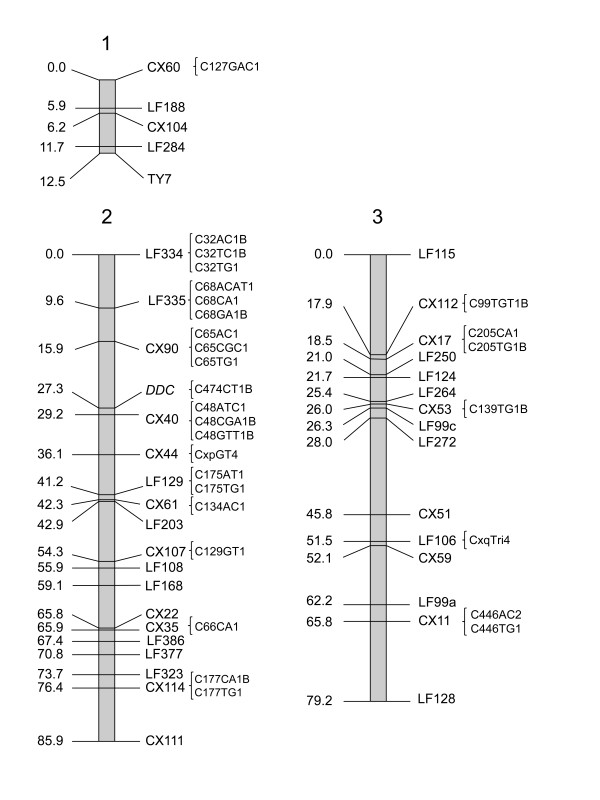
**Chromosome locations of the 29 SSRs used to screen the NDJ BAC library**. SSR markers indicated by brackets '{' were extracted and developed from genome assembly supercontig sequences anchored as indicated to loci from the existing linkage map [48,49]. Map distances are given in Kosambi centiMorgans.

To further assess the quality of the library, we prepared and screened DNA extractions representing each of the 144 plate pools with 29 simple sequence repeat (SSR) markers representing all three linkage groups (Figure 3). The number of positive plate pools for each SSR ranged from 0 to 29, resulting in a mean of 8.7 positive plate pools per screen. Only one of the SSR markers (C134AC1) did not amplify in any of the plate pools. Nevertheless, a gene sequence (CPIJ007110) on the same supercontig (3.134), ~80 kb downstream, amplified in 12 plate pools. In addition to the SSRs, we screened the plate pools with primers designed to amplify sequences within exons of four genes previously determined to have a role in reproductive diapause in *Cx. pipiens sensu stricto *(*s.s*.) [42,43]. The number of positive plate pools for the gene sequences ranged from three to 23, resulting in a mean of 10.8 positive plate pools per gene (Table 2). The size distribution of the four individual clones selected by probing with radiolabeled markers is similar to the size distribution of the library (Figure 2A). The overall mean number of positive plate pools for the 29 SSRs and five genes used to screen the library was 9.0, indicating that the NDJ BAC library represents ~9 BAC clones per marker across the *Cx. quinquefasciatus *genome, assuming that only one BAC clone per 384-well plate pool contains the target sequence.

Detailed genetic and genomic studies among the *Cx. pipiens *complex could provide valuable insights into the molecular genetic mechanisms influencing important traits such as vector competence, insecticide resistance, and reproductive diapause. Despite morphological similarities and their ability to form hybrid populations, species within the complex differ in several life history traits. For example, *Cx. quinquefasciatus *requires a blood meal prior to laying eggs (anautogenous) and is unable to enter diapause and overwinter in cold climates. *Cx. pipiens *and *Cx. pipiens pallens *also are anautogenous but adult females are able to enter reproductive diapause and survive winter in temperate climates, and *Cx. pipiens molestus *is able to lay eggs without taking a blood meal (autogenous) but does not enter diapause [45-47]. Presently, detailed molecular analyses of these traits are limited by the fragmented genome assembly. Fingerprinting, end-sequencing and physical assembly of the NDJ BAC library would likely facilitate the construction of a more complete genome sequence assembly by serving as a template for genome finishing, including gap-filling, as well as providing resources to enable the assignment of the individual superscaffolds to their respective chromosome position via *in situ *hybridization. In summary, the NDJ BAC library provides a valuable resource for marker development, positional cloning, and genome sequence assembly enhancement for *Cx. quinquefasciatus *thus helping to advance genome studies in *Cx. pipiens *complex mosquitoes.

## Library availability

The NDJ BAC library is available to researchers through the Clemson University Genomics Institute (see *Culex pipiens *library CPQLBa at http://www.genome.clemson.edu/).

## Competing interests

The authors declare that they have no competing interests.

## Authors' contributions

PVH drafted the manuscript and helped design probes for library screening. AM performed the initial genetic mapping and provided the pupae used in library construction. CAS constructed the library, estimated insert sizes and assisted in writing the manuscript. BD and DDL designed probes for library screening. BD performed plate pool screening. The project was conceived and supervised by DWS. All authors approved of the final version.
